# Peer Review of “Finding Potential Adverse Events in the Unstructured Text of Electronic Health Care Records: Development of the Shakespeare Method”

**DOI:** 10.2196/31551

**Published:** 2021-08-11

**Authors:** Haiyan Yu

**Affiliations:** 1 Center for Data and Decision Sciences Chongqing University of Posts and Telecommunications Chongqing China

*This is a peer-review report submitted for the paper “Finding Potential Adverse Events in the Unstructured Text of Electronic Health Care Records: Development of the Shakespeare Method”*

## Round 1 Review

### General Comments

This paper [1] investigated the new and increasing rates of adverse events (AEs) in unstructured text in electronic health records (EHRs). The topic is interesting. The authors used the Shakespeare method to identify attributed and unattributed potential AEs with EHRs. This method would be a useful supplement to AE reporting and surveillance. Although I believe that the topic of the study is very relevant, I have some concerns related to the theoretical background of the study. Specific major and minor comments are listed below.

### Specific Comments

#### Major Comments

1. What is the accuracy of the new method, the Shakespeare method, for identifying attributed and unattributed potential AEs? The previous study showed the process of this method in the literature [2]. This paper did not mention the accuracy of the new method.

#### Minor Comments

1. Too many keywords. I would suggest that the authors reduce some of the keywords.
2. In the “Conclusions” subsection, I would suggest the paragraphs be reorganized to improve them.

## Abbreviations

AE: adverse event
EHR: electronic health record
